# *ARSA* Variants Associated With Cognitive Decline and Long‐Term Preservation of Motor Function in Metachromatic Leukodystrophy

**DOI:** 10.1002/jimd.70072

**Published:** 2025-08-02

**Authors:** Shanice Beerepoot, Daphne H. Schoenmakers, Francesca Fumagalli, Samuel Groeschel, Ludger Schöls, Raphael Schiffmann, Sheila Wong, Odile Boespflug‐Tanguy, Caroline Sevin, Yann Nadjar, Annette Bley, Fanny Mochel, Morten A. Horn, Cristina Baldoli, Sara Locatelli, Holger Hengel, Lucia Laugwitz, Carla E. M. Hollak, Volkmar Gieselmann, Marjo S. van der Knaap, Nicole I. Wolf

**Affiliations:** ^1^ Amsterdam Leukodystrophy Center, Department of Child Neurology Emma Children's Hospital, Amsterdam UMC, Location Vrije Universiteit Amsterdam the Netherlands; ^2^ Amsterdam Neuroscience Cellular & Molecular Mechanisms, Vrije Universiteit Amsterdam the Netherlands; ^3^ Nierkens and Lindemans Group, Princess Máxima Center for Pediatric Oncology Utrecht the Netherlands; ^4^ Platform “Medicine for Society”, Department of Endocrinology and Metabolism Amsterdam UMC, Location University of Amsterdam Amsterdam the Netherlands; ^5^ San Raffaele Telethon Institute for Gene Therapy (SR‐TIGET), IRCCS San Raffaele Scientific Institute Milan Italy; ^6^ Pediatric Immunohematology Unit and BMT Program IRCCS San Raffaele Scientific Institute Milan Italy; ^7^ Neurology and Neurophysiology Unit IRCCS San Raffaele Scientific Institute Milan Italy; ^8^ Neuropaediatrics, General Paediatrics, Diabetology, Endocrinology and Social Paediatrics University of Tübingen, University Hospital Tübingen Tübingen Germany; ^9^ Department of Neurology and Hertie‐Institute for Clinical Brain Research University of Tübingen Tübingen Germany; ^10^ German Center for Neurodegenerative Diseases (DZNE) Tübingen Germany; ^11^ Texas Christian University Fort Worth Texas USA; ^12^ Department of Pediatrics and Adolescent Medicine Hong Kong Children's Hospital Hong Kong China; ^13^ AP‐HP, Service de neuropédiatrie, French Reference Center for Leukodystrophies and Université Paris Cité, UMR 1141, INSERM, NeuroDiderot, Hopital Robert Debré Paris France; ^14^ TIDU GENOV, Institut du Cerveau et de la Moelle Épinière, ICM, Inserm UMR 1127, CNRS UMR 7225, Sorbonne Université Paris France; ^15^ Department of Neuropediatrics, French Reference Center for Leukodystrophies Bicêtre Hospital Paris France; ^16^ Neuro‐Metabolism Unit, Reference Center for Lysosomal and Metabolic Neurological Diseases, Department of Neurology Pitié‐Salpêtrière University Hospital, AP‐HP Paris France; ^17^ Department of Pediatrics, Leukodystrophy Clinic University Medical Center Hamburg Eppendorf Hamburg Germany; ^18^ Reference Center for Neurometabolic Diseases and Leukodystrophies, Department of Medical Genetics Pitié‐Salpêtrière University Hospital, AP‐HP Paris France; ^19^ Department of Neurology Oslo University Hospital Oslo Norway; ^20^ Neuroradiology Unit IRCCS San Raffaele Scientific Institute Milan Italy; ^21^ Institute for Biochemistry and Molecular Biology, Medical Faculty, University of Bonn Bonn Germany; ^22^ Center for Neurogenomics and Cognitive Research, Integrative Neurophysiology, Amsterdam Neuroscience, Vrije Universiteit Amsterdam the Netherlands

**Keywords:** *ARSA* gene, arylsulfatase A, genetic association studies, hematopoietic stem cell transplantation, metachromatic leukodystrophy

## Abstract

Patients with metachromatic leukodystrophy (MLD) show variable motor and cognitive decline. The *ARSA* variants c.256C>T, p.(Arg86Trp), c.257G>A, p.(Arg86Gln) and c.542T>G, p.(Ile181Ser) are associated with predominantly cognitive decline. This multinational study analyzed MLD onset type, presenting signs/symptoms, cognitive function, gross motor function, central motor tract involvement, MRI severity score, peripheral neuropathy, and survival of 47 patients (three homozygous for c.256C>T and five, twelve and 27 compound heterozygous for c.256C>T, c.257G>A, or c.542T>G and another *ARSA* variant, respectively). Eleven underwent hematopoietic stem cell transplantation (HSCT). Onset was late‐juvenile (46.8%) or adult (44.7%) with predominantly cognitive decline (*n* = 40/41 symptomatic patients). At diagnosis, untreated patients typically retained independent walking (100%), sparing of central motor tracts (87.5%), and absence of demyelinating neuropathy (95.5%), which persisted in follow‐up for most (76.5%, 71.4%, and 64.7%, respectively). Early‐juvenile onset and rapid motor decline occurred only in patients compound heterozygous for c.256C>T and a severe second variant (*n* = 4), showing central motor tract involvement at diagnosis. One untreated and one treated patient died of disease progression, and another from HSCT complications. All other treated patients retained independent walking, and four of five tested normal cognitive function. Median MRI severity score remained lower in treated (13) than untreated patients (25). The phenotype of c.256C>T carriers depends on the severity of the second *ARSA* variant. Patients harboring c.257G>A or c.542T>G show late‐juvenile or adult onset with cognitive decline and preserved motor function, usually associated with sparing of central motor tracts. In these patients, cognitive function and MRI severity score should be preferred treatment outcomes.

## 1 Introduction

Metachromatic leukodystrophy (MLD, MIM #250100) is a neurometabolic disorder caused by biallelic disease‐causing variants in *ARSA* (MIM*607574), resulting in deficiency of the lysosomal enzyme arylsulfatase A (ASA) and subsequent (lyso)sulfatides accumulation in the peripheral and central nervous system [1]. The clinical course of MLD is characterized by deterioration of gross and fine motor skills and cognitive function, ultimately leading to a severely disabled state and premature death [2, 3, 4]. The rate of deterioration largely depends on the age of symptom onset (ASO), with a younger ASO resulting in a faster disease progression than an older ASO [2, 3]. MLD has been classified into four onset types: late‐infantile (ASO: < 30 months), early‐juvenile (ASO: 2.5–6 years), late‐juvenile (ASO: 7–16 years), and adult (ASO: > 16 years) [5, 6]. This classification is used for defining study populations and determining eligibility for disease‐modifying treatments, either with allogeneic hematopoietic stem cell transplantation (HSCT) or autologous hematopoietic stem cell gene therapy (arsa‐cel). HSCT is considered the standard treatment for pre‐ and early‐symptomatic patients with late‐juvenile or adult‐onset MLD and has shown limited efficacy in early‐juvenile MLD. Arsa‐cel is approved for use in pre‐symptomatic late‐infantile and early‐juvenile patients, as well as in early‐symptomatic early‐juvenile cases [4, 7, 8, 9]. The type of symptoms at onset has been recognized as an additional clinical predictor of disease progression, with patients presenting with pure cognitive and behavioral symptoms exhibiting slower deterioration than patients presenting with motor symptoms. Some patients even retain normal motor function for decades without therapeutic intervention [4, 10]. These natural history data are important, not only for counseling and therapeutic decision making, but also for the evaluation of treatment effects in pre‐ and post‐registration studies, which often include motor function as a primary outcome [11]. In addition, identification of prognostic factors are essential to interpret results from future newborn screening in MLD [12].

Currently, the cause of clinical variation in MLD is not fully understood. A genotype–phenotype correlation has been proposed based on the predicted functionality of disease‐causing *ARSA* variants: variants resulting in very low or absent residual ASA activity (0‐alleles) are linked to early‐onset MLD phenotypes, while variants resulting in higher activity (R‐alleles) are associated with late‐onset phenotypes. Exact activity thresholds are not well defined and depend on assay sensitivity [4, 6, 10, 13, 14, 15, 16]. Recently, Trinidad et al. proposed an MLD phenotype matrix, which classifies patients as late‐infantile, juvenile, adult, or asymptomatic based on variant classification as severe, moderate, or mild [17]. Potential associations between disease‐causing *ARSA* variants and the type of symptoms at onset have also been reported, mainly in late‐juvenile and adult MLD. Most of these reports comprise isolated cases [18], except for compound heterozygosity for the common R‐allele c.542T>G, p.(Ile181Ser) and other disease‐causing *ARSA* variants, which have repeatedly been linked to a cognitive onset [4, 10, 19, 20, 21, 22]. R‐allele c.256C>T, p.(Arg86Trp) in homozygous state or compound heterozygous state with another disease‐causing *ARSA* variant has been associated with no or only mild peripheral neuropathy [4]. Considering the current scarcity of genotype–phenotype associations in MLD and the increasing need for disease prediction in research and clinical settings, we aimed to further characterize clinical and MRI phenotypes of patients harboring the following variants—c.256C>T, c.257G>A p.(Arg86Gln), which alters the same amino acid as c.256C>T, or c.542T>G—and to examine the effect of treatment with HSCT in these patients.

## 2 Methods

### 2.1 Standard Protocol Approvals and Patient Consents

This cross‐sectional study was approved by the local ethics committees of each participating hospital. Written informed consent was obtained from the patients or their guardians.

### 2.2 Subjects

Patients were included by the treating physician in 10 international centers (Amsterdam UMC, Baylor Scott & White Research Institute, Hong Kong Children's Hospital, Robert Debré Hospital, Bicêtre Hospital, Pitié‐Salpêtrière University Hospital, University Medical Center Hamburg Eppendorf, University Hospital Tübingen, Oslo University Hospital, and IRCCS San Raffaele Scientific Institute). These centers span eight countries, with the European sites affiliated with the MLD Initiative—Disease Registry for MLD. Patients were eligible for the study if they had (1) a genetically and biochemically confirmed diagnosis of MLD [6], or, if biochemical confirmation was not performed in family screening, identification of the same disease‐causing *ARSA* variants as in an affected sibling, and (2) at least one of the identified *ARSA* variants included c.256C>T, p.(Arg86Trp), c.257G>A, p.(Arg86Gln) or c.542T>G, p.(Ile181Ser) in *ARSA*. Note that the numbering used here follows the GenBank NM_000487.5 reference sequence, which differs by two amino acids compared to the original X52151.1 sequence and Uniprot entries, where c.256C>T, p.(Arg86Trp), and c.257G>A, p.(Arg86Gln) are referred to as c.250C>T, p.(Arg84Trp), and c.251G>A, p.(Arg84Gln), respectively, and c.542T>G, p.(Ile181Ser) as c.536T>G, p.(Ile179Ser).

### 2.3 Data Collection

Clinical data on patient and treatment characteristics and test results at diagnosis and follow‐up (FU, latest clinical evaluation) were obtained as part of standard clinical care or prospective natural history studies (Table S1) [4, 10]. For each data point, FU in years was calculated from diagnosis for all patients, and disease duration was calculated from symptom onset for symptomatic patients. The functional severity of the disease‐causing *ARSA* variants identified in this study was classified based on Trinidad et al. [17]. Based on this classification, c.256C>T and c.257G>A would be considered moderate variants, and c.542T>G as a mild variant.

### 2.4 Assessments

**FIGURE 1:**
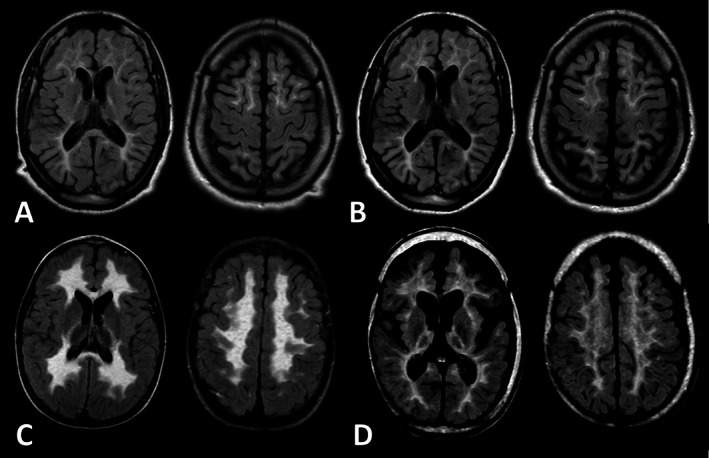
MRI assessment of central motor tract involvement. Transverse FLAIR images illustrating central motor tract involvement at the posterior limb of the internal capsule (left) and the white matter under the Rolandic area (right). Upper panel: Images from a patient homozygous for the c.256C>T, p.(Arg86Trp) variant with sparing of the central motor tracts, showing nearly normal signal intensity in the white matter of the corticospinal tracts under the Rolandic area at diagnosis at age 30 years (A) and follow‐up (FU) at age 37 years (B), despite surrounding abnormal white matter with increased signal intensity and abnormal signal in the internal capsule. Lower panel: Images from a patient compound heterozygous for c.256C>T, p.(Arg86Trp), and c.465+1G>A, p.(?) without sparing of the central motor tracts, displaying significantly increased signal intensity in the white matter of the corticospinal tracts under the Rolandic area at diagnosis at age 4 years (C) and FU at age 14 years (D), similar to the surrounding abnormal white matter and abnormal internal capsule.

Assessments included MLD onset type, presenting signs and symptoms, gross motor function, cognitive function, involvement of the motor tracts on brain MRI, severity of MRI abnormalities, presence of peripheral neuropathy, and survival. MLD onset type was determined based on the ASO for symptomatic patients or based on the MLD onset type of a symptomatic sibling for the presymptomatic patients, according to previously published definitions [5, 6, 23]. Presenting signs and symptoms were considered as the first problems noticed by the patient/family and were translated into five dichotomous variables: “presymptomatic,” “cognitive decline,” “psychiatric manifestations including behavioral abnormalities” [24], “impaired gross motor function,” and “other presentation.” Since different neuropsychological tests were used and subscores were not available, three cognitive levels based on full‐scale intelligence quotient (FSIQ) scores were defined: “no to borderline impaired cognitive function (FSIQ: ≥ 70),” “mildly impaired cognitive function (FSIQ: 50–69),” and “severely impaired cognitive function (FSIQ: ≤ 49)” [25]. In addition, patients with a FSIQ higher than 85 were analyzed separately as this threshold is suggested as treatment eligibility criterion [26]. Gross motor function was scored according to the validated Gross Motor Function Classification in MLD (GMFC‐MLD) [27]. As rapid decline of motor function has been associated with central motor tract involvement [28, 29], we hypothesized that long‐term motor function preservation in these patients might correlate with sparing of the central motor tracts in MRI. Available neuroimaging was evaluated by local radiologists or (pediatric) neurologists, who assessed whether the white matter of the corticospinal tracts under the Rolandic area was visually “spared” or “not spared” on FLAIR images [29], independent of involvement of the posterior limb of the internal capsule (Figure 1). Severity of MRI abnormalities was scored using the MLD MRI severity score according to Eichler et al. [30]. Presence of peripheral neuropathy was determined based on nerve conduction study (NCS) and electromyography (EMG) reports stating whether the patient exhibited a demyelinating peripheral neuropathy and/or signs of axonal degeneration according to locally used criteria and reference values. Survival was calculated in years from birth to death for deceased patients and to last clinical evaluation for patients alive.

### 2.5 Statistical Methods

All patients were included in the analyses of assessments performed at diagnosis. Analyses of FU assessments were performed separately for patients treated with HSCT and those untreated. Categorical parameters were described by counts and percentages, while ordinal and continuous parameters were reported using median, interquartile range (iqr), and minimum–maximum ranges due to non‐normality, as determined graphically and with the Shapiro–Wilk test. Univariable analyses were conducted using Chi‐Square Test, Fisher's exact test, Mann–Whitney *U* Test, or Kruskal–Wallis multiple comparison with Benjamini‐Hochberg stepwise adjustment, as appropriate. All statistical tests were two‐tailed, and *p* values < 0.05 were considered statistically significant. All analyses were performed using R version 4.3.2 (RStudio: Integrated Development for R. RStudio Inc., Boston, MA).

## 3 Results

### 3.1 Patient Demographics

The study population consisted of 47 patients with MLD, of whom 23 (48.9%) were male. There were eight sibling pairs. Eight patients (17.0%) carried the c.256C>T variant, three of them in homozygous state. Twelve patients (25.5%) were compound heterozygous for c.257G>A and another variant, and 27 (57.4%) were compound heterozygous for the c.542T>G variant. The most common second disease‐causing *ARSA* variant in trans was a severe second variant, most often c.465 + 1G>A, p.(?) (0‐allele), found in 72.3% of the patients. Individual patient demographics are shown in Table S2, including information on the functional severity classification of the second variant. Median age at diagnosis was 19 years (iqr: 12–31, range: 1–42 years). Most patients (*n* = 41, 87.3%) were symptomatic with a median ASO of 13 years (iqr: 8–26, range: 4–33 years). Eleven patients (23.4%) received HSCT at a median age of 17 years (iqr: 9–27, range: 4–35 years). Patient demographics for the separate *ARSA* variant groups are shown in Table 1, which revealed no statistically significant differences between the groups, except that homozygosity was observed exclusively in patients with the *c.256C>T* variant, not for the two other variants studied. There was only one patient who had an otherwise common mild *ARSA* variant, c.1283C>T, in trans.

**TABLE 1:** Patient demographics.

Variable^a^	c.256C>T	c.257G>A	c.542T>G	*p* ^b^
	(*n* = 8)	(*n* = 12)	(*n* = 27)	
Male	3 (37.5)	6 (50.0)	14 (51.9)	0.851
*ARSA* homozygosity	3 (37.5)	0 (0)	0 (0)	**0.003**
Severe second variant^c^	4 (50.0)	8 (66.7)	22 (81.5)	0.190
Residual ASA activity measurement	8 (100)	9 (75.0)	25 (93.6)	0.159
Urinary sulfatide measurement	3 (37.5)	4 (33.3)	15 (55.5)	0.402
Symptomatic	7 (87.5)	11 (91.7)	23 (85.1)	1.000
Age of onset, y	6 (4–21)	10 (8–22)	18 (12–28)	0.200
Age at diagnosis, y	11 (4–33)	15 (11–29)	25 (15–30)	0.513
HSCT	1 (12.5)	3 (25.0)	7 (25.9)	0.801
Age at HSCT, y	4 (NA)	28 (17–32)	18 (14–27)	NA^d^

*Note:* Significant *p* values are indicated in bold.

Abbreviations: HSCT = allogeneic hematopoietic stem cell transplantation; NA = not assessed; y = years.

^a^ Values indicate median (interquartile range) or number (percent) unless otherwise stated.

^b^ *p* values were obtained with a Fisher's exact test or Kruskal–Wallis test as appropriate.

^c^ Functional severity of the second *ARSA* variants is indicated based on Trinidad et al. [17].

^d^ Differences in age at HSCT were not assessed due to the small patient numbers.

### 3.2 Assessments

Table 2 summarizes assessments at the time of diagnosis for patients with the c.256C>T, c.257G>A, or c.542T>G variant on one allele separately. Follow‐up data for untreated and treated patients are also presented separately for each variant group. While the second variants are functionally distinct, grouping was performed to investigate how patients with one of the c.256C>T, c.257G>A, or c.542T>G variants present, irrespective of the second allele. Table S2 provides a detailed overview of all patients, including the variants in trans. Table S3 presents median disease duration at FU assessments for untreated patients across various outcomes, allowing for comparisons of disease duration differences between patients with distinct outcomes.

**TABLE 2:** Measurements at diagnosis for all patients and at follow‐up for untreated and treated patients separately.

Variable^a^	c.256C>T (*n* = 8)	c.257G>A (*n* = 12)	c.542T>G (*n* = 27)	Total (*n* = 47)
MLD onset type^b^	0/4/1/3	0/0/8/4	0/0/13/14	0/4/22/21
Presenting symptoms	7 (8)	11 (12)	23 (27)	41 (47)
Cognitive decline	6 (7)	11 (11)	23 (23)	40 (41)
Psychiatric manifestations	4 (7)	10 (11)	19 (23)	33 (41)
Impaired gross motor function	1 (7)	0 (11)	0 (23)	1 (41)
Other additional symptoms	1 (7)	2 (11)	0 (23)	3 (41)
Cognitive function^c^	0/0/1	3/4/1	7/3/2	10/7/4
Cognitive function FU UT^c^	0/0/4	0/1/0	1/1/4	1/2/8
Cognitive function FU TT^c^	0/0/1^e^	2/1/0	3/0/0	5/1/1^f^
Gross motor function^d^	3/2/0/0/0/1/0	11/1/0/0/0/0/0	27/0/0/0/0/0/0	41/3/0/0/0/1/0
Gross motor function FU UT^d^	3/0/0/0/0/0/4	3/3/0/0/0/2/0	14/3/1/1/0/0/0	20/6/1/1/0/2/4
Gross motor function FU TT^d^	0/0/0/0/0/0/1	3/0/0/0/0/0/0	5/1/0/0/0/0/0	8/1/0/0/0/0/1
Involvement of the motor tracts	4 (7)	0 (10)	3 (25)	7 (42)
Involvement of the motor tracts FU UT	3 (7)	1 (4)	2 (10)	6 (21)
Involvement of the motor tracts FU TT	1^e^ (1)	0 (3)	2 (7)	3 (11)
MRI severity	19 (17–22)	18 (13–20)	19 (15–22)	19 (15–22)
MRI severity FU UT	26 (22–31)	23 (22–24)	25 (21–26)	25 (21–26)
MRI severity FU TT	33^e^ (NA)	13 (13–15)	13 (10–21)	13 (13–21)
Demyelinating neuropathy	0 (8)	0 (8)	4 (9)	4 (29)
Demyelinating neuropathy FU UT	2 (7)	0 (1)	4 (9)	6 (17)
Demyelinating neuropathy FU TT	0^e^ (1)	0 (3)	0 (3)	0 (7)
Axonal degenerative neuropathy	2 (8)	2 (8)	1 (13)	5 (29)
Axonal degenerative neuropathy FU UT	3 (7)	1 (1)	2 (9)	6 (17)
Axonal degenerative neuropathy FU TT	0^e^ (1)	1 (3)	1 (3)	2 (7)
Deceased UT	1 (7)	0 (9)	0 (20)	3 (36)
Deceased TT	1^e^ (1)	0 (3)	1 (7)	2 (11)
Survival from birth UT	42 (23–47)	28 (19–36)	35 (25–38)	34 (24–40)
Survival from birth TT	9^e^ (NA)	38 (24–41)	15 (17–30)	17 (14–34)

Abbreviations: FU = follow‐up; HSCT = allogeneic hematopoietic stem cell transplantation; NA = not assessed; TT = treated; UT = untreated; y = years.

^a^ Values indicate median (interquartile range) or number (number of patients analyzed) unless otherwise stated.

^b^ Number of patients diagnosed with a late‐infantile/early‐juvenile/late‐juvenile/adult MLD phenotype.

^c^ Cognitive function was only determined in patients with available full‐scale intelligence quotient scores and categories are presented as “no to borderline impaired cognitive function”/“mildly impaired cognitive function”/“severely impaired cognitive function.”

^d^ Gross motor function are categories are presented as “GMFC‐MLD = 0”/“GMFC‐MLD = 1”/“GMFC‐MLD = 2”/“GMFC‐MLD = 3”/“GMFC‐MLD = 4”/“GMFC‐MLD = 5”/“GMFC‐MLD = 6.”

^e^ Patient who died due to disease progression after HSCT failure.

### 3.3 MLD Onset Type

**FIGURE 2:**
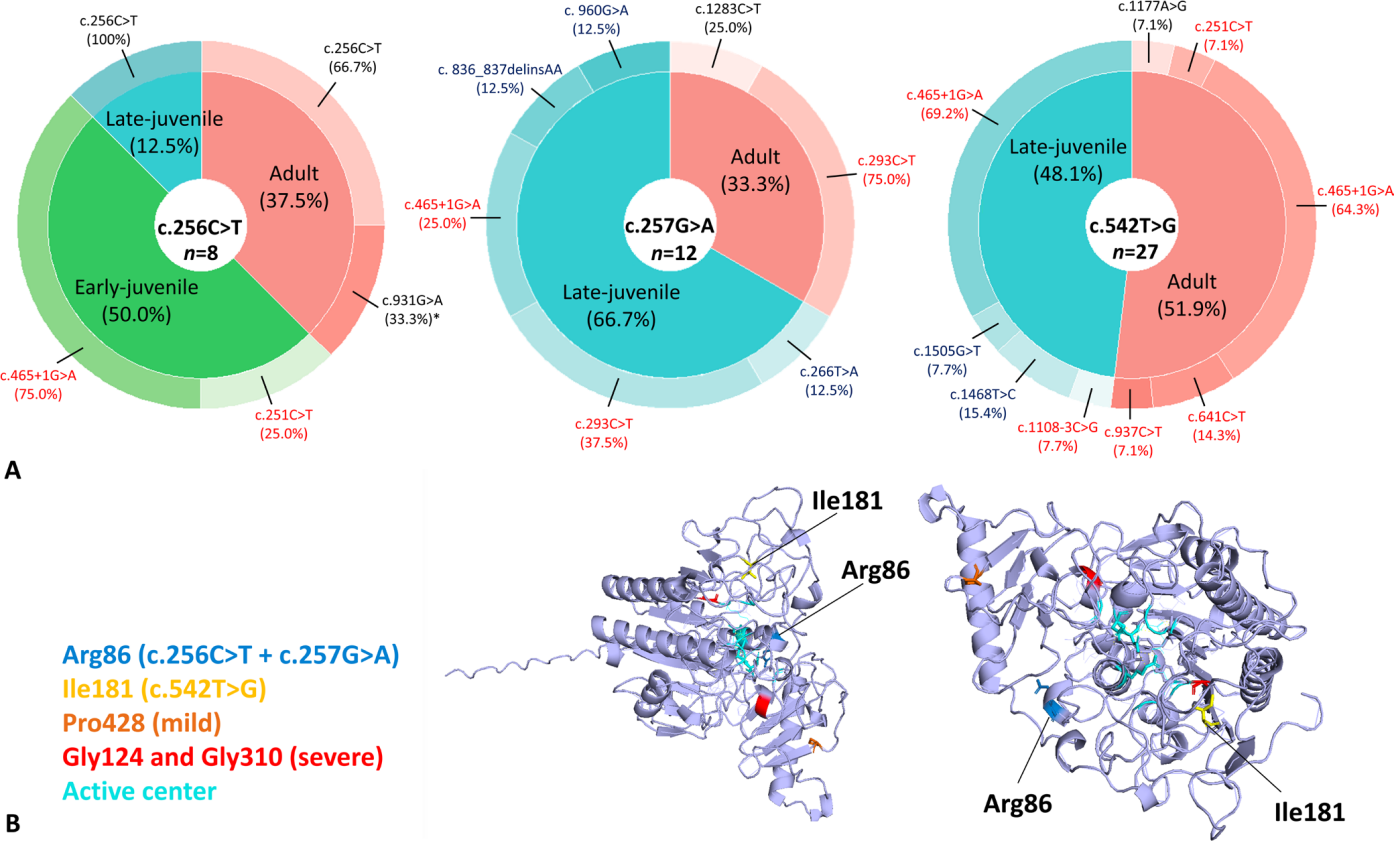
Distribution and severity of *ARSA* variants identified in this study and location of affected amino acid residues. (A) Donut chart illustrating the proportion of patients with MLD onset types within the c.256C>T, p.(Arg86Trp), c.257G>A, p.(Arg86Gln), and c.542T>G, p.(Ile181Ser) groups. The distribution of the second *ARSA* variants per MLD onset type is shown across these groups. Functional severity of the second *ARSA* variants is indicated based on Trinidad et al., with known severe variants in red, known mild or moderate variants in black, and variants with unknown functional severity in blue. Asterisks (*) denote conflicting interpretations of functional severity. (B) 3D model of the ASA monomer (Protein Data Bank 1AUK, visualized using the PyMOL Molecular Graphics System, Version 3.0.3 Schrödinger LLC [31]) depicting the locations of amino acid residue Arg86 (blue, affected by the c.256C>T and c.257G>A variants) and Ile181 (yellow, affected by the c.542T>G variant) and three other commonly affected amino acid residues in MLD: Pro428, commonly affected in patients with late‐juvenile and adult MLD, is orange. Gly124 and Gly310, affected in patients with late‐infantile MLD, are red. Amino acid residues forming the active center of ASA are highlighted in teal. Helices, β‐sheets, and loops are represented as ribbons, arrows, and threads, respectively.

Most patients had either adult (*n* = 21, 44.7%) or late‐juvenile (*n* = 22, 46.8%) MLD. None of the patients had late‐infantile MLD. Four patients (10.3%) had early‐juvenile MLD, all of whom were compound heterozygous for c.256C>T and a severe second variant according to Trinidad et al. [16]. No severe second variants were found in trans in the late‐juvenile and adult patients carrying the c.256C>T variant, whereas severe variants *in trans* were common in the late‐juvenile and adult patients carrying the c.257G>A or c.542T>G variant. Figure 2 depicts the distribution and functional severity of second *ARSA* variants among the MLD onset types within the three groups.

### 3.4 Presenting Signs and Symptoms

**FIGURE 3:**
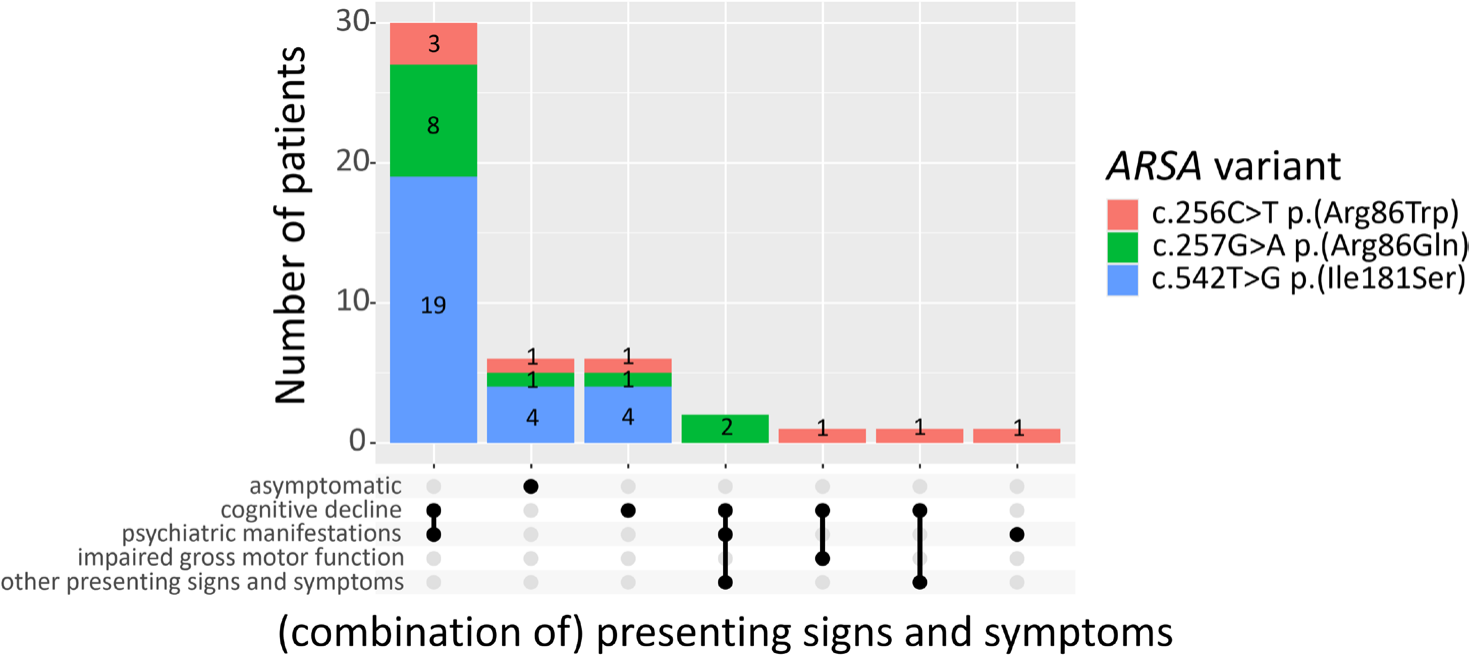
Overview of presenting signs and symptoms. Black dots represent individual presenting signs and symptoms or combinations of them (multiple black dots connected). The total bar length indicates the total number of patients with each combination. Separate colored bars show the number of patients with each combination per *ARSA* variant included in the study. Other presenting signs and symptoms were impaired fine motor skills in two patients (one early‐juvenile patient compound heterozygous for c.256C>T, p.(Arg86Trp) and one late‐juvenile patient compound heterozygous for c.257G>A, p.(Arg86Gln), both harboring a severe second variant in trans) and spasticity in one late‐juvenile patient (compound heterozygous for c.257G>A, p.(Arg86Gln) and a severe second variant in trans).

Six patients, diagnosed because of symptomatic family members, were asymptomatic at presentation. Of the 41 symptomatic patients, 40 presented with cognitive decline, either alone (*n* = 6, 15.0%) or with additional symptoms (*n* = 34, 85.0%), with psychiatric manifestations being the most prevalent (*n* = 32). Figure 3 displays the distribution of all combinations of presenting signs and symptoms. One patient, homozygous for c.256C>T, presented exclusively with psychiatric manifestations at age 31 years. Only one patient (2.4%), compound heterozygous for c.256C>T and a severe second variant in trans, presented with impaired gross motor function (balance problems at the age of 3.9 years), in addition to cognitive decline.

### 3.5 Cognitive Function

**FIGURE 4:**
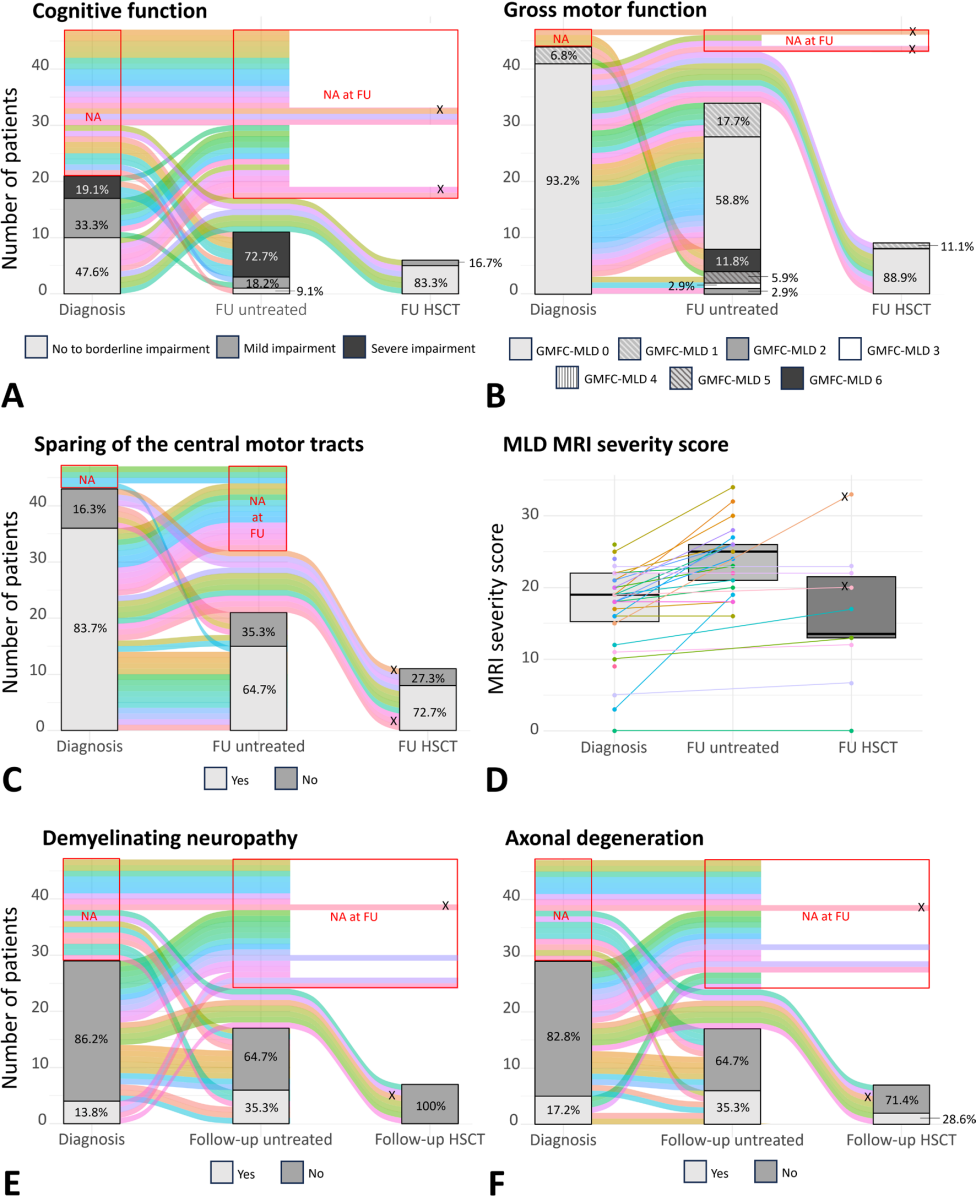
Overview of measurements at diagnosis and follow‐up, with separated analysis for untreated and HSCT‐treated patients. (A–C, E, F) Bar graphs show patient counts and percentages by category at diagnosis and follow‐up (FU). Colored lines connect individual patients to their respective categories, while unconnected lines within the red boxes indicate no assessments at diagnosis (NA) and/or FU (NA at FU). X's denote the two patients who died due to HSCT complications (pink) or MLD progression after unsuccessful HSCT due to engraftment failure (orange). (D) Box plots display median and iqr at diagnosis and FU, with dots representing individual patients and lines connecting their measurements at diagnosis and FU. (A) Cognitive function categories: No to borderline impairment (FSIQ ≥ 70), mild impairment (FSIQ 50–69), and severe impairment (FSIQ ≤ 49). (B) Gross Motor Function according to the GMFC‐MLD scale with score 0 indicating normal locomotion and score 6 indicating loss of all gross motor function; no patients scored 4. (C) Central motor tracts sparing: Yes/No, based on whether the white matter of the corticospinal tracts under the Rolandic area was visually “spared” or “not spared”, independent of involvement of the posterior limb of the internal capsule. (D) MLD MRI severity score with score 0 indicating no MRI abnormalities and score 34 most severe MRI abnormalities: The median annual increase in MRI severity score was 1.0 in the untreated group and 0.3 in the treated group. (E) Demyelinating neuropathy and (F) Signs of axonal degeneration on nerve conduction studies and electromyography: Yes/No, according to locally used criteria and reference values.

FSIQ was measured at diagnosis for 21/47 patients. Four patients (19.1%) had severe, seven (33.3%) had mild, and ten (47.6%) had no to borderline cognitive impairment (Figure 4A). Of these ten patients, five had an FSIQ above 85 and were still presymptomatic.

At FU, 11 untreated patients were tested (Figure 4A). Eight had severe cognitive impairment (all but one with an FSIQ ≤ 40; three already had an FSIQ ≤ 40 at diagnosis). Two had mild cognitive impairment: in one patient, FSIQ dropped from 67 to 54 over 3.5 years, and the other had an FSIQ of 61 after 14.8 years with no prior measurement. One patient, compound heterozygous for c.542T>G, had an FSIQ of 92 after 4 years, despite reported cognitive decline at diagnosis without an initial FSIQ measurement. There were no differences between genotype groups or second variant severity.

### 3.6 Gross Motor Function and Sparing of the Central Motor Tracts

At diagnosis, 41/44 (93.2%) had a GMFC‐MLD score of 0, while three (6.8%) had a score of 1, indicating mild gait abnormalities (Figure 4B). All patients with mild motor signs were compound heterozygous for a severe second variant and showed central motor tract involvement. Of all 43 patients with neuroimaging data at diagnosis, 36 (83.7%) demonstrated sparing of the central motor tracts (Figure 4C), more commonly in those harboring the c.257G>A (100%) or c.542T>G (88.0%) variant, compared to those harboring the c.256C>T variant (50.0%). In the latter groups, involvement of the motor tracts was associated with a severe variant in trans in most patients (Table S2).

During FU, the majority of the untreated patients (26/34, 76.5%) retained independent walking ability with a GMFC‐MLD score of 0 (*n =* 20) or 1 (*n* = 6) (Figure 4B). Six patients (17.7%) lost all locomotion, including all three who had mild motor impairment (score 1) at diagnosis. Among these, all four patients who were compound heterozygous for c.256C>T and a severe second variant deteriorated to GMFC‐MLD 6, while all patients homozygous for c.256C>T maintained GMFC‐MLD 0. In patients harboring the c.257G>A or c.542T>G variant, a severe second variant was not associated with motor decline (*p* = 0.407). However, the only two patients with GMFC‐MLD 5 were compound heterozygous for c.257G>A and a severe second variant, with one of them presenting a score of 1 at diagnosis. Gross motor decline was associated with central motor tract involvement at diagnosis (*p* < 0.001) and FU (*p* = 0.006) in all groups. Central motor tract involvement was observed only in patients with a severe second *ARSA* variant. Importantly, repeated neuroimaging in 21 untreated patients revealed that, except for two cases, all patients with initial central motor tract sparing maintained this sparing at FU, with a maximum FU duration of 21 years (Figure 4C).

There was no significant difference in disease duration between patients who experienced motor decline and those who did not (*p* = 0.493), nor between those with and without central motor tract sparing (*p* = 0.533).

### 3.7 MRI Severity

The median MLD MRI severity score at diagnosis was 19 (iqr: 15–22, range: 0–26, Figure 4D) and was comparable among patients harboring one of the three variants (*p* = 0.746), regardless of a severe second variant in trans (*p* = 0.531).

In most untreated patients (88.2%), MLD MRI severity score was increased at FU, resulting in a median score of 25 (iqr: 21–26, range: 16–34, Figure 4D). The median increase per year was 1.0, and both total increase and increase rate were comparable among patients carrying one of the three variants (*p* = 0.679 and *p* = 0.452, respectively). Patients carrying a severe second variant in trans tended to have higher MRI severity scores (median: 26) and a greater annual increase (median: 0.8) than those carrying a mild or moderate second variant (median score: 23, median increase: 0.3, *p* = 0.100). Patients carrying a second variant of unknown functional severity exhibited the highest annual increase (median: 1.5, *p* = 0.044).

### 3.8 Presence of Peripheral Neuropathy

Twenty‐nine patients underwent NCS at diagnosis. Four (13.8%) were diagnosed with demyelinating neuropathy, while five (17.2%) different patients showed signs of axonal involvement without fulfilling the local diagnostic criteria for a demyelinating neuropathy (Figure 4E,F). One of these later was diagnosed with demyelinating neuropathy at FU.

At FU, among the 17 untreated patients tested, the proportion of patients diagnosed with demyelinating neuropathy and those with axonal degeneration both increased to 35.3% (Figure 4E,F). Half of these patients were diagnosed with both. There were no differences between genotype groups or second variant severity.

### 3.9 Survival

One (2.8%) untreated patient, who was compound heterozygous for c.256C>T and a severe second variant, died of disease progression 4.8 years after onset, at the age of 10.3 years. The remaining 36 (97.3%) untreated patients were alive, with a median survival of 34.2 years (iqr: 24.9–39.8, range: 10.3–52.1). While survival did not vary between genotype groups, the presence of a severe second variant was linked to a significantly shorter survival duration (*p* < 0.001). This was attributed to a younger age at the time of the study, as death occurred in only one untreated patient.

### 3.10 Effects of HSCT

Eleven patients were treated with HSCT (Table 3). Except for two patients (#257‐11 and #542‐8) with a second *ARSA* variant of unknown functional severity according to Trinidad et al. [17], all of them carried a severe second variant in trans. Two late‐juvenile patients (#257‐11 and #542‐1) and three adult patients (#257‐3, #542‐3, and #542‐21) were presymptomatic at the time of treatment. One early‐juvenile patient (#256‐3) exhibited cognitive impairment and impaired fine motor skills, with onset occurring 6 months before treatment. Three late‐juvenile patients (#542‐8, #542‐22, and #542‐24) and two adult patients (#257‐2 and #542‐6) displayed cognitive and psychiatric signs and symptoms, with onset ranging from 1.5 to 7.0 years prior to treatment. Except for patient #256‐3, whose GMFC‐MLD score was unknown, all patients had a GMFC‐MLD score of 0 at the time of treatment. All patients with available FSIQ data (#257‐2, #257‐3, #542‐1, #542‐3, #542‐8, and #542‐21) exhibited no to borderline impaired cognitive function. The central motor tracts were spared in all patients except #256‐3, #542‐22, and #542‐24. Patients #542‐3, #542‐6, and #542‐22 had a demyelinating neuropathy, whereas patients #256‐3, #257‐2, #257‐3, #257‐11, and #542‐21 did not.

**TABLE 3:** Clinical characteristics and outcomes of individual treated patients before and after HSCT.

Patient ID	Symptoms (ASO)	Onset type	Age HSCT^a^	GMFC‐MLD (treatment FU^a^)	Cognitive function^b^ (treatment FU^a^)	Involvement of the motor tracts (treatment FU^a^)	MRI severity score (treatment FU^a^)	Demyelinating peripheral neuropathy (treatment FU^a^)	Axonal degeneration (treatment FU^a^)	Deceased (treatment FU^a^)
256‐3	Cognitive decline, impaired fine motor skills (4)	EJ	4	NA/6 (5)	NA/Severe (3)	Yes/Yes (5)	15/33 (5)	No/No (5)	No/No (5)	Yes (5) Disease progression after engraftment failure
257‐2	Cognitive decline, psychiatric manifestations (25)	A	28	0/0 (9)	No/No (3)	Spared/Spared (6)	12/17 (6)	No/No (3)	No/No (3)	No (10)
257‐3	Presymptomatic	A	35	0/0 (8)	No/No (5)	Spared/Spared (8)	10/13 (8)	No/No (2)	No/Yes (2)	No (11)
257‐11	Presymptomatic	LJ	6	0/0 (4)	NA/Mild (3)	Spared/Spared (3)	11/12 (3)	No/No (4)	No/No (4)	No (4)
542‐1	Presymptomatic	LJ	7	0/0 (11)	No/No (11)	Spared/Spared (11)	0/0 (11)	NA/No (8)	NA/No (8)	No (9)
542‐3	Presymptomatic	A	26	0/0 (11)	No/NA (−)	Spared/Spared (8)	12/13 (8)	Yes/No (11)	No/Yes (11)	No (11)
542‐6	Cognitive decline, psychiatric manifestations (29)	A	31	0/0 (2)	NA/NA (−)	Spared/Spared (2)	12/13 (2)	Yes/NA (−)	No/NA (−)	No (< 1)
542‐8	Cognitive decline, psychiatric manifestations (10)	LJ	13	0/NA (−)	No/NA (−)	Spared/Spared (< 1)	19/20 (< 1)	NA/NA (−)	NA/NA (−)	Yes (< 1) HSCT related
542‐21	Presymptomatic	A	29	0/0 (< 1)	No/No (< 1)	Spared/Spared (< 1)	5/7 (< 1)	No/NA (−)	No/NA (−)	No (< 1)
542‐22	Cognitive decline, psychiatric manifestations (11)	LJ	18	0/0 (< 1)	NA/NA (−)	Yes/Yes (< 1)	22/22 (< 1)	Yes/NA (−)	No/NA (−)	No (< 1)
542‐24	Cognitive decline, psychiatric manifestations (10)	LJ	15	0/1 (< 1)	NA/No (< 1)	Yes/Yes (< 1)	23/23 (< 1)	NA/No (< 1)	NA/No (< 1)	No (< 1)

*Note:* Clinical characteristics and outcomes of individual treated patients are shown. For GMFC‐MLD, cognitive function, involvement of the motor tracts, MRI severity score, demyelinating peripheral neuropathy, and axonal degeneration, the outcome at diagnosis and at latest follow‐up is presented. For outcomes at latest follow‐up, the treatment follow‐up is indicated between brackets. Survival is calculated from treatment to death for deceased patients and to last clinical evaluation for living patients. < 1 indicate that the last evaluation was within the first year after HSCT.

Abbreviations: A = adult; ASO = age of symptom onset; EJ = early‐juvenile; FU = follow‐up; HSCT = allogeneic hematopoietic stem cell transplantation; LI = late‐infantile; LJ = late‐juvenile; NA = not assessed.

^a^ Age and treatment follow‐up are given in years.

^b^ Cognitive function categories are presented as “no” indicating “no to borderline impaired cognitive function”; “mild” indicating “mildly impaired cognitive function.”

One patient (#542‐8) died from HSCT complications at the age of 13 years, one patient (#256–3) at the age of 9.4 years due to disease progression, 5 years after unsuccessful HSCT due to engraftment failure. The remaining nine patients were still alive at a median age of 29 years (iqr: 16–37, range: 9.5–44), in stable neurological condition, following initial disease progression shortly after HSCT in some cases (#257‐3 and #542‐24).

Presymptomatic patient #257–3, who had an MRI score of 10 before HSCT, developed cognitive and psychiatric signs and symptoms within the first year after treatment, with an FSIQ drop from 87 to 73. The four other presymptomatic patients (#257‐11, #542‐1, #542‐3, and #542‐21) remained without symptoms at ages 9.5, 15.5, 37.4, and 29.0 years, respectively, with stable neuropsychological testing. Symptomatic patient #542‐24 progressed to a GMFC‐MLD score of 1 immediately after HSCT, while all other surviving patients maintained a GMFC‐MLD score of 0.

In all patients whose central motor tracts were spared at the time of treatment, these tracts remained spared at FU. The MRI severity score remained stable in three patients (33.3%), including a score of 0 in patient #542‐1 after 8.5 years post‐HSCT at age 15.5 years. The score increased by three or fewer points in five patients (55.6%) and by five points in one patient (#257‐2, 11.1%), resulting in a median MRI severity score of 13 (iqr: 12–17, range: 0–23). The median increase per year was 0.3. None of the seven patients tested were diagnosed with demyelinating neuropathy, but two (28.6%) developed signs of axonal degeneration.

## 4 Discussion

This study compares clinical phenotypes of 47 patients with MLD harboring c.256C>T, p.(Arg86Trp), c.257G>A, p.(Arg86Gln) or c.542T>G, p.(Ile181Ser) in *ARSA* and examines the effect of HSCT on the disease course in eleven of them. In line with previous reports, our data reveal that the phenotype of these patients is characterized by mainly cognitive decline, often accompanied by psychiatric manifestations, including behavioral problems.

All patients harboring the *ARSA* variants c.257G>A or c.542T>G in compound heterozygous state, or c.256C>T in homozygous state, had a late‐juvenile or adult MLD onset of cognitive decline. Even without treatment, most patients retained independent walking, with sparing of the central motor tracts up to 30 years after onset. Notably, more than two‐thirds of these patients carried a severe second *ARSA* variant in trans. Still, the age of onset was variable in this patient group, ranging from 6 to 33 years. Our findings are consistent with previous reports on untreated patients with these genotypes [4, 10, 19, 20, 21, 22, 32], although these studies did not analyze the involvement of the central motor tracts. Why these tracts are spared and remain so is not known.

A severe second *ARSA* variant according to Trinidad et al. was associated with a more severe clinical presentation only in patients harboring the c.256C>T variant in a compound heterozygous state [17]. These patients exhibited an early‐juvenile MLD onset of cognitive decline, followed by rapid motor deterioration. MRI showed corticospinal tract involvement already at diagnosis in all of them. This observation aligns with previous findings that central motor region involvement based on FA values in the pyramidal tracts or spatial distribution of demyelination load predicts a more severe disease progression [28, 29]. Our study suggests that central motor tract involvement is associated with the *ARSA* genotype.

Nine of the eleven HSCT‐treated patients were alive at the time of the study, all retaining the ability to walk independently, and four out of the five tested had normal cognitive function. Additionally, MRI severity scores at FU were lower in the HSCT group than in the untreated group. While this difference may partly reflect lower initial scores at diagnosis, the median annual increase in MRI severity scores was also reduced in the HSCT group. Typically, MRI severity scores increase during the first year after HSCT and stabilize or decrease in subsequent years [33], resulting in larger differences between treated and untreated patients after longer FU. It is important to note that two treated patients died following HSCT, resulting in a survival rate of 81.8%. One death was due to complications, illustrating the inherent risks of HSCT, while the other occurred due to disease progression after engraftment failure, reflecting the natural disease course in patients compound heterozygous for c.256C>T and a severe second variant. In contrast, all but one of the untreated patients remained alive, with a median survival of 34 years. However, longer follow‐up is needed to fully assess the long‐term benefits of HSCT, particularly with regard to motor function and survival, given the slow progression of the disease associated with these *ARSA* variants.

Our study exemplifies that disease prediction based on genotype in cases of compound heterozygosity involving a 0‐ and R‐allele, or combination of severe and mild or moderate functional severity as defined by Trinidad et al. is not straightforward. This complexity is evident in the equal distribution of the 0‐allele/severe variant c.465+1G>A, p.(?) among late‐juvenile and adult patients in the c.542T>G group, where ASO ranged from 6 to 27 years. Although residual ASA activity was not assessed in this study, it may provide additional predictive insight. Santhanakumaran et al. reported higher residual ASA activity in patients with cognitive disease onset compared to those with motor onset; they were unable to distinguish late‐juvenile from adult onset [16]. A close correlation between levels of residual ASA activity and ASO has yet to be established [6]. It is possible that (epi)genetic or environmental factors also influence the phenotype of individual patients, even within families [8].

Biffi et al. hypothesized that the severity of peripheral neuropathy might be an additional tool for predicting clinical severity, suggesting that no or mild demyelinating neuropathy indicates less severe disease progression [14]. While the majority of patients in this study, which focused on specific genotypes associated with less severe disease progression, exhibited no or mild demyelinating neuropathy, consistent with this hypothesis, normal conduction velocities were also observed in the patients with an early MLD onset and rapid gross motor deterioration.

Interestingly, we did not identify the c.257G>A and c.542T>G variants in homozygous state in this study, neither in previously published MLD patients [6, 17, 34, 35]. Therefore, the classification of c.257G>A as a moderate variant, as proposed by Trinidad et al., may need to be reconsidered as a mild variant [17]. Additionally, no patients compound heterozygous for any combination of the variants c.256C>T, c.257G>A, and c.542T>G have been identified, despite their relatively high frequency [4, 6, 17, 34, 36]. Another intriguing finding is that only one patient in our cohort was compound heterozygous with the mild c.1283C>T, p.(Ser428Phe) as the second *ARSA* variant, despite its relatively high frequency. One possible explanation for the absence of these genotypes is misdiagnosis as dementia due to late adulthood and absent motor signs. This is supported by a patient compound heterozygous for c.542T>G and c.1283C>T who presented around the age of 60 years with a frontal syndrome, without signs of polyneuropathy or motor impairment. Only NCS indicated mild demyelinating neuropathy [37, 38]. However, these genotypes have not been reported in large dementia cohorts so far [39]. Another possibility for the absence of combinations of the three studied variants (c.256C>T, c.257G>A, and c.542T>G) is that these genotypes may lack clinical consequences. The MLD phenotype matrix by Trinidad et al. suggests that individuals with mild/mild (including c.542T>G/c.542T>G) or mild/moderate (including c.542T>G/c.256C>T) genotype combinations may be asymptomatic or have very late or subtle symptom onset [17]. Further support comes from gnomAD (v4.1.0, accessed 09/2024), which identifies a presumed healthy individual carrying the c.542T>G variant in a homozygous state.

Analyzing these three *ARSA* variants and related phenotypes demonstrates that detailed phenotype information on disease‐causing *ARSA* variants (not limited to age of onset but describing long‐term disease course and associated instrumental parameters) is essential to interpret results from newborn screening and to advance phenotype prediction to aid decisions regarding treatment timing and mode [16]. Although inclusion of later‐onset disorders in NBS is debated, detailed genotype–phenotype correlations can guide individualized decisions and help define actionable cases. In addition, genotype–phenotype associations are crucial for the proper interpretation of treatment effects. Long‐term preservation of motor function is typical for c.257G>A and c.542T>G compound heterozygous and c.256C>T homozygous patients, limiting the value of motor function as a primary outcome in (experimental) treatments concerning this patient group. Instead, preventing severe cognitive decline should be considered the preferred primary outcome and treatment goal. Additionally, MRI severity score seems to be a promising long‐term marker of treatment effects.

Several study limitations deserve mention. Due to study design, we were unable to collect exact times of clinical and radiological deterioration, such as the age of loss of ambulation, limiting Kaplan–Meier analyses. Nevertheless, the long FU periods and the homogeneity in clinical and radiological features of patients compound heterozygous for either c.257G>A or c.542T>G, regardless of the second variant in trans, as well as the significant differences between these patients and those compound heterozygous for c.256C>T with a severe second variant, add robustness to our findings. Some test results were obtained using different methods and reference values, inherent to an international multicenter study spanning two decades. Consequently, residual ASA enzyme activity was not assessed in this study, results of peripheral neuropathy were based on local interpretations, and results of FSIQ were categorized rather than using exact outcomes, which may have induced information bias. This limitation underscores the need for harmonized test protocols in rare diseases often requiring international patient cohorts to achieve sufficient numbers.

As with most retrospective studies, missing data could potentially lead to selection bias. This issue is particularly relevant for cognitive function, as FSIQ testing is rarely performed on patients with severe cognitive impairment. Selective non‐testing may account for the discrepancy between reports of cognitive decline and the relatively high proportion of patients with no to borderline impairment of cognitive function. In addition, patients with leukodystrophies might face behavioral and daily living challenges that are more severe than their cognitive functioning would suggest [40]. Finally, the study had only a small number of treated patients with a relatively short FU period, who were, on average, younger and less affected than those in the untreated group. This is particularly notable given the slow and late progression of the disease in patients with this genotype. As a result, while the findings suggest differences in cognitive function and MRI severity, definitive conclusions about the HSCT effects cannot be drawn.

In conclusion, despite genetic heterogeneity due to the second variant in trans, patients with MLD harboring the c.257G>A or c.542T>G variant on one allele in *ARSA* typically exhibit slow disease progression characterized by mainly cognitive decline and long‐term preservation of motor function. The early onset of rapid motor decline in patients harboring the c.256C>T variant on one allele depends on the severity of the second variant in trans. Those who are homozygous for c.256C>T or carry another mild second variant tend to present similarly to patients with c.257G>A or c.542T>G. Patients who are compound heterozygous for any combination of the variants c.256C>T, c.257G>A, c.542T>G, or another mild variant may be asymptomatic or experience very late or subtle symptom onset. This prognosis should be weighed against the risks of HSCT. Furthermore, to evaluate treatment effects in clinical trials in pre‐ and post‐registration studies, cognitive function and MRI severity scores should be preferred as treatment outcomes in patients with these genotypes, as motor function, peripheral nerve function, and survival can be retained for decades. In this cohort, treated patients demonstrated stable cognitive function and MRI severity scores after HSCT. However, larger studies are needed to assess treatment efficacy specifically in patients primarily experiencing cognitive decline. Further research is needed to explore whether other mild *ARSA* variants are associated with similar phenotypes and to better understand their long‐term clinical course.

## Author Contributions

**Shanice Beerepoot:** drafting/revision of the manuscript for content, including medical writing for content, major role in the acquisition of data, study concept or design, analysis or interpretation of data. **Daphne H. Schoenmakers:** drafting/revision of the manuscript for content, including medical writing for content, major role in the acquisition of data. **Francesca Fumagalli:** drafting/revision of the manuscript for content, including medical writing for content, major role in the acquisition of data. **Samuel Groeschel:** drafting/revision of the manuscript for content, including medical writing for content, major role in the acquisition of data. **Ludger Schöls:** drafting/revision of the manuscript for content, including medical writing for content, major role in the acquisition of data. **Raphael Schiffmann:** drafting/revision of the manuscript for content, including medical writing for content, major role in the acquisition of data. **Sheila Wong:** drafting/revision of the manuscript for content, including medical writing for content, major role in the acquisition of data. **Odile Boespflug‐Tanguy:** drafting/revision of the manuscript for content, including medical writing for content, major role in the acquisition of data. **Caroline Sevin:** drafting/revision of the manuscript for content, including medical writing for content, major role in the acquisition of data. **Yann Nadjar:** drafting/revision of the manuscript for content, including medical writing for content, major role in the acquisition of data. **Annette Bley:** drafting/revision of the manuscript for content, including medical writing for content, major role in the acquisition of data. **Fanny Mochel:** drafting/revision of the manuscript for content, including medical writing for content, major role in the acquisition of data. **Morten A. Horn:** drafting/revision of the manuscript for content, including medical writing for content, major role in the acquisition of data. **Cristina Baldoli:** analysis or interpretation of data. **Sara Locatelli:** major role in the acquisition of data. **Holger Hengel:** drafting/revision of the manuscript for content, including medical writing for content, major role in the acquisition of data. **Lucia Laugwitz:** drafting/revision of the manuscript for content, including medical writing for content, analysis or interpretation of data. **Carla E. M. Hollak:** drafting/revision of the manuscript for content, including medical writing for content, major role in the acquisition of data. **Volkmar Gieselmann:** drafting/revision of the manuscript for content, including medical writing for content; analysis or interpretation of data. **Marjo S. van der Knaap:** drafting/revision of the manuscript for content, including medical writing for content, major role in the acquisition of data. **Nicole I. Wolf:** drafting/revision of the manuscript for content, including medical writing for content, major role in the acquisition of data; study concept or design.

## Ethics Statement

This study was approved by the local ethics committees of each participating hospital.

## Consent

Written informed consent was obtained from the patients or their guardians.

## Conflicts of Interest

Nicole I. Wolf is advisor and/or co‐investigator for trials in MLD (Shire/Takeda, Orchard, Evidera) and other leukodystrophies (Ionis, PassageBio, Vigil Neuro), with all payments to the institution. Annette Bley is coinvestigator/advisor for trials in MLD (Shire/Takeda, Orchard). Caroline Sevin is an advisor and/or investigator for trials in MLD (Shire/Takeda, Orchard). Francesca Fumagalli is an investigator of gene therapy clinical trials for MLD sponsored by Orchard Therapeutics, the license holder of Libmeldy, and acts as a consultant for the ad hoc Advisory Board of Orchard Therapeutics and Takeda. Carla E. M. Hollak is involved in premarketing studies with Sanofi, Protalix, and Idorsia, for which financial arrangements are made with Amsterdam UMC Research BV. These activities are unrelated to the content of this manuscript. Ludger Schöls served as a consultant for Vico Therapeutics, Vigil Neuroscience, and Novartis unrelated to MLD and the manuscript. Samuel Groeschel received an Institutional Research Support from Shire plc and Orchard, and is an advisor and coinvestigator for trials in MLD (Shire/Takeda, Orchard) without personal payments. Yann Nadjar received a research grant from Orchard. The other authors declare no conflicts of interest.

## Supporting information


**Data S1:** Supporting Information.

## Data Availability

Pseudonymized individual data in this article will be made available on reasonable request from a qualified investigator, after approval of the Institutional Review Board of the hospitals that own the data in question.
