# Supplementary material for: ARSA Variants Associated With Cognitive Decline and Long‐Term Preservation of Motor Function in Metachromatic Leukodystrophy
Source: J Inherit Metab Dis. 2025 Aug 2;48(5):e70072. doi: 10.1002/jimd.70072 (PMC12317651; doi:10.1002/jimd.70072)
Supplement: Supplementary file 1 — Data S1: Supporting Information. [file JIMD-48-0-s001.docx]

# Supplementary material

**Supplementary Table 1:** **Measurements collected in the study at diagnosis and latest clinical evaluation**

| **Clinical variable** | **Coding** |
| --- | --- |
| Sex at birth | Male; Female |
| Age of symptom onset | Years |
| Age at diagnosis | Years |
| Presenting symptoms | Qualitative description |
| Treatment performed | Yes, No |
| Age at treatment if applicable | Years |
| Neurological exam at diagnosis | Qualitative description |
| Neurological exam at latest clinical evaluation  (FU duration since diagnosis and disease duration since symptom onset) | Qualitative description (years) |
| GMFC-MLD at diagnosis | Score from 0 – 6 |
| GMFC-MLD at latest clinical evaluation (FU duration since diagnosis and disease duration since symptom onset) | Score from 0 – 6 (years) |
| Disease-causing *ARSA* variant allele 1 | Qualitative description |
| Disease-causing *ARSA* variant allele 2 | Qualitative description |
| Residual ASA activity in leukocytes at diagnosis | Unit and reference values according to local laboratory protocol |
| ASA activity in leukocytes at latest clinical evaluation  (FU duration since diagnosis and disease duration since symptom onset) | Unit and reference values according to local laboratory protocol (years) |
| Urinary sulfatide excretion at diagnosis | Unit and reference values according to local laboratory protocol |
| Urinary sulfatide excretion at latest clinical evaluation  (FU duration since diagnosis and disease duration since symptom onset) | Unit and reference values according to local laboratory protocol (years) |
| MRI: MRI severity score at diagnosis | Score from 0 – 34 |
| MRI: MRI severity score at latest clinical evaluation  (FU duration since diagnosis and disease duration since symptom onset) | Score from 0 – 34 (years) |
| MRI: Sparing of the motor tracts at diagnosis | Yes, No |
| MRI: Sparing of the motor tracts at latest clinical evaluation  (FU duration since diagnosis and disease duration since symptom onset) | Yes, No (years) |
| Full-scale intelligence quotient score at diagnosis | Score from 0 - 150 |
| Full-scale intelligence quotient score at latest clinical evaluation  (FU duration since diagnosis and disease duration since symptom onset) | Score from 0 - 150 (years) |
| Nerve conduction velocity sensory nerves at diagnosis | Meter per second if available for ulnar nerve, median nerve, and sural nerve |
| Nerve conduction velocity motor nerves at diagnosis | Meter per second if available for ulnar nerve, median nerve, peroneal nerve, and tibial nerve |
| Demyelinating peripheral according to the locally used criteria and reference values at diagnosis | Yes, No |
| Signs of axonal degeneration according to the locally used criteria and reference values at diagnosis | Yes, No |
| Nerve conduction velocity sensory nerves at latest clinical evaluation  (FU duration since diagnosis and disease duration since symptom onset) | Meter per second if available for ulnar nerve, median nerve, and sural nerve (years) |
| Nerve conduction velocity motor nerves at latest clinical evaluation  (FU duration since diagnosis and disease duration since symptom onset) | Meter per second if available for ulnar nerve, median nerve, peroneal nerve, and tibial nerve (years) |
| Demyelinating peripheral according to the locally used criteria and reference values at latest clinical evaluation  (FU duration since diagnosis and disease duration since symptom onset) | Yes, No (years) |
| Signs of axonal degeneration according to the locally used criteria and reference values at latest clinical evaluation  (FU duration since diagnosis and disease duration since symptom onset) | Yes, No (years) |
| Deceased? | Yes, No |
| Age at death if applicable | Years |
| Cause of death | Disease progression; treatment complications; other / unknown |
| Survival from birth until death or latest clinical evaluation | Years |

**Supplementary Table 2: Clinical characteristics and outcomes of individual patients**

| **Patient ID** | **cDNA variant 1** | **cDNA variant 2^a^** | **ASO^c^** | **Age^c^ at diagnosis** | **Presenting symptoms at diagnosis** | **Onset type** | **HSCT (age^c^)** | **GMFC-MLD (disease duration^c^)** | **Cognitive function^b^ (disease duration^c^)** | **Involvement of the motor tracts (disease duration^c^)** | **MRI severity score (disease duration^c^)** | **Demyelinating peripheral neuropathy (disease duration^c^)** | **Axonal degeneration (disease duration^c^)** | **Deceased (survival^c^)** |
| --- | --- | --- | --- | --- | --- | --- | --- | --- | --- | --- | --- | --- | --- | --- |
| 256-1 | c.256C>T | **c.251C>T** | 4 | 5 | Cognitive decline, psychiatric manifestations | EJ | No | 1 / 6 (15) | NA / Severe (9) | Yes / Yes (11) | 19 / 32 (11) | No / Yes (18) | Yes / Yes (18) | No (25) |
| 256-2 | c.256C>T | **c.465+1G>A** | 4 | 4 | Cognitive decline, impaired balance | EJ | No | 1 / 6 (15) | Severe / Severe (7) | Yes / Yes (10) | 21 / 30 (10) | No / Yes (14) | No / Yes (14) | No (21) |
| 256-3 @ | c.256C>T | **c.465+1G>A** | 4 | 4 | Cognitive decline, impaired fine motor skills | EJ | Yes, failed (4) | NA / 6 (5) | NA / Severe (3) | Yes / Yes (5) | 15 / 33 (5) | No / No (5) | No / No (5) | Yes (9) disease progression |
| 256-4 | c.256C>T | c.256C>T | 31 | 32 | Psychiatric manifestations | A | No | 0 / 0 (7) | NA / NA (-) | Spared / Spared (14) | 17 / 18 (14) | No / No (15) | No / No (15) | No (48) |
| 256-5 | c.256C>T | c.931G>A* | 33 | 37 | Cognitive decline | A | No | NA / 6 (15) | NA / NA (-) | Spared / Spared (6) | 22 / 26 (6) | No / No (11) | No / No (11) | No (50) |
| 256-6 # | c.256C>T | c.256C>T | 11 | 17 | Cognitive decline, psychiatric manifestations | LJ | No | 0 / 0 (27) | NA / Severe (29) | NA / Spared (27) | NA / 25 (27) | No / No (27) | No / No (27) | No (42) |
| 256-7 @ | c.256C>T | **c.465+1G>A** | 5 | 6 | Cognitive decline, psychiatric manifestations | EJ | No | NA / 6 (4) | NA / Severe (4) | Yes / Yes (4) | 25 / 34 (4) | No / No (4) | Yes / Yes (4) | Yes (10) disease progression |
| *256-8 #* | *c.256C>T* | *c.256C>T* | *-* | *42* | *Presymptomatic* | *A* | *No* | *0 / 0 (2)* | *NA / NA (-)* | *Spared / Spared (1)* | *16 / 16 (1)* | *No / No (1)* | *No / No (1)* | *No (46)* |
| 257-1 $ | c.257G>A | **c.293C>T** | NA | 33 | Cognitive decline, psychiatric manifestations | A | No | 0 / 5 (19) | NA / NA (-) | Spared / NA (-) | 26 / NA (-) | NA / NA (-) | NA / NA (-) | No (52) |
| 257-2 $ | c.257G>A | **c.293C>T** | 25 | 27 | Cognitive decline, psychiatric manifestations | A | Yes (28) | 0 / 0 (12) | No / No (6) | Spared / Spared (9) | 12 / 17 (9) | No / No (6) | No / No (6) | No (38) |
| *257-3 $* | *c.257G>A* | ***c.293C>T*** | *-* | *35* | *Presymptomatic* | *A* | *Yes (35)* | *0 / 0 (8)* | *No / No (5)* | *Spared / Spared (8)* | *10 / 13 (8)* | *No / No (2)* | *No / Yes (2)* | *No (44)* |
| 257-4 | c.257G>A | **c.293C>T** | 8 | 10 | Cognitive decline, psychiatric manifestations, spasticity | LJ | No | 1 / 5 (19) | Mild / NA (-) | NA / NA (-) | NA / NA (-) | No / NA (-) | No / NA (-) | No (28) |
| 257-5 | c.257G>A | **c.293C>T** | 7 | 13 | Cognitive decline, psychiatric manifestations | LJ | No | 0 / 1 (10) | Mild / NA (-) | Spared / Spared (10) | 20 / 23 (10) | No / NA (-) | No / NA (-) | No (19) |
| 257-6 | c.257G>A | **c.293C>T** | 8 | 10 | Cognitive decline, psychiatric manifestations | LJ | No | 0 / 1 (3) | Mild / NA (-) | Spared / NA (-) | 20 / NA (-) | No / NA (-) | Yes / NA (-) | No (11) |
| 257-7 | c.257G>A | c.1283C>T | 32 | 40 | Cognitive decline, psychiatric manifestations | A | No | 0 / 1 (9) | Severe / NA  (-) | Spared / Spared (9) | 22 / 23 (9) | No / NA (-) | No / NA (-) | No (41) |
| 257-8 | c.257G>A | c.836_837delinsAA | 14 | 20 | Cognitive decline, psychiatric manifestations | LJ | No | 0 / 0 (8) | Mild / NA (-) | Spared / Spared (8) | 18 / 20 (8) | No / NA (-) | Yes / NA (-) | No (22) |
| 257-9 | c.257G>A | **c.465+1G>A** | 8 | 11 | Cognitive decline, psychiatric manifestations, impaired fine motor skills | LJ | No | 0 / 0 (24) | NA / NA (-) | NA / NA (-) | NA / NA (-) | NA / NA (-) | NA / NA (-) | No (36) |
| 257-10 | c.257G>A | **c.465+1G>A** | 12 | 17 | Cognitive decline, psychiatric manifestations | LJ | No | 0 / 0 (15) | NA / NA (-) | Spared / Yes (15) | 16 / 27 (15) | NA / No (13) | NA / Yes (13) | No (32) |
| *257-11* | *c.257G>A* | *c.960G>A* | *-* | *6* | *Presymptomatic* | *LJ* | *Yes (6)* | *0 / 0 (4)* | *NA / Mild (3)* | Spared / Spared *(3)* | *11 / 12 (3)* | *No / No (4)* | *No / No (4)* | *No (10)* |
| 257-12 | c.257G>A | c.266T>A | 8 | 11 | Cognitive decline | LJ | No | 0 / NA (-) | No / NA (-) | Spared / NA (-) | 16 / NA (-) | NA / NA (-) | NA / NA (-) | No (11) |
| *542-1* | *c.542T>G* | ***c.465+1G>A*** | *-* | *1* | *Presymptomatic* | *LJ* | *Yes (7)* | *0 / 0 (11)* | *No / No (11)* | Spared / Spared *(11)* | *0 / 0 (11)* | *NA / No (8)* | *NA / No (8)* | *No (16)* |
| *542-2* | *c.542T>G* | *c.1177A>G* | *-* | *26* | *Presymptomatic* | *A* | *No* | *0 / 0 (12)* | *NA / NA (-)* | Spared / Spared *(11)* | *19 / 23 (11)* | *No / No (9)* | *No / No (9)* | *No (38)* |
| *542-3 %* | *c.542T>G* | ***c.465+1G>A*** | *-* | *25* | *Presymptomatic* | *A* | *Yes (26)* | *0 / 0 (11)* | *No / NA (-)* | Spared / Spared *(8)* | *12 / 13 (8)* | *Yes / No (11)* | *No / Yes (11)* | *No (37)* |
| 542-4 % | c.542T>G | **c.465+1G>A** | 18 | 19 | Cognitive decline, psychiatric manifestations | A | No | 0 / 1 (16) | No / NA (-) | Spared / NA (-) | 22 / NA (-) | NA / NA (-) | NA / NA (-) | No (36) |
| 542-5 | c.542T>G | **c.465+1G>A** | 6 | 8 | Cognitive decline, psychiatric manifestations | LJ | No | 0 / 2 (3) | Mild / NA (-) | Yes / Yes (3) | 19 / 21 (3) | NA / Yes (3) | NA / No (3) | No (12) |
| 542-6 | c.542T>G | c.1505G>T | 29 | 30 | Cognitive decline, psychiatric manifestations | A | Yes (31) | 0 / 0 (2) | NA / NA (-) | Spared / Spared (2) | 12 / 13 (2) | Yes / NA (-) | No / NA (-) | No (31) |
| 542-7 | c.542T>G | **c.465+1G>A** | NA | 30 | Cognitive decline, psychiatric manifestations | LJ | No | 0 / 0 (4) | NA / NA (-) | Spared / NA (-) | 18 / NA (-) | NA / No (2) | NA / Yes (2) | No (37) |
| 542-8 & | c.542T>G | c.1468T>C | 10 | 12 | Cognitive decline, psychiatric manifestations | LJ | Yes (13) | 0 / NA (-) | No / NA (-) | Spared / Spared (3) | 19 / 20 (3) | NA / NA (-) | NA / NA (-) | Yes (13) HSCT related |
| *542-9 &* | *c.542T>G* | *c.1468T>C* | *-* | *8* | *Presymptomatic* | *LJ* | *No* | *0 / 0 (7)* | *NA / No (4)* | *Spared / Spared (7)* | *3 / 19 (7)* | *NA / No (4)* | *NA / No (4)* | *No (26)* |
| 542-10 | c.542T>G | **c.251C>T** | 32 | 32 | Cognitive decline, psychiatric manifestations | A | No | 0 / 0 (11) | NA / NA (-) | Spared / Spared (11) | 18 / 24 (11) | NA / Yes (11) | NA / No (11) | No (47) |
| 542-11 + | c.542T>G | **c.465+1G>A** | 7 | 21 | Cognitive decline, psychiatric manifestations | LJ | No | 0 / 0 (31) | NA / Severe (23) | Spared / Spared (31) | 21 / 26 (31) | NA / Yes (31) | NA / No (31) | No (41) |
| 542-12 + | c.542T>G | **c.465+1G>A** | 14 | 16 | Cognitive decline, psychiatric manifestations | LJ | No | 0 / 1 (21) | NA / Severe (9) | Spared / Spared (19) | 18 / 26 (19) | NA / No (19) | NA / No (19) | No (36) |
| 542-13 | c.542T>G | **c.465+1G>A** | 17 | 25 | Cognitive decline, psychiatric manifestations | A | No | 0 / 0 (16) | Mild / NA (-) | Spared / NA (-) | 24 / NA (-) | No / NA (-) | No / NA (-) | No (34) |
| 542-14 | c.542T>G | **c.465+1G>A** | 12 | 12 | Cognitive decline, psychiatric manifestations | LJ | No | 0 / 3 (7) | Severe / Severe (10) | Yes / Yes (9) | 20 / 28 (9) | No / Yes (10) | No / Yes (10) | No (24) |
| 542-15 | c.542T>G | **c.1108-3C>G** | 14 | 17 | Cognitive decline, psychiatric manifestations | LJ | No | 0 / 0 (6) | Severe / Severe (9) | NA / Yes (9) | NA / 26 (9) | No / No (9) | No / No (9) | No (24) |
| 542-16 = | c.542T>G | **c.465+1G>A** | 19 | 38 | Cognitive decline, psychiatric manifestations | A | No | 0 / 0 (19) | NA / NA (-) | Spared / NA (-) | 22 / NA (-) | NA / NA (-) | NA / NA (-) | No (39) |
| 542-17 = | c.542T>G | **c.465+1G>A** | 27 | 36 | Cognitive decline, psychiatric manifestations | A | No | 0 / 0 (9) | NA / NA (-) | NA / NA (-) | NA / NA (-) | NA / NA (-) | NA / NA (-) | No (37) |
| 542-18 | c.542T>G | **c.937C>T** | 19 | 28 | Cognitive decline | A | No | 0 / 0 (10) | Mild / NA (-) | Spared / NA (-) | 20 / NA (-) | NA / NA (-) | NA / NA (-) | No (29) |
| 542-19 ¥ | c.542T>G | **c.261C>T** | 30 | 32 | Cognitive decline | A | No | 0 / 0 (4) | NA / NA (-) | Spared / NA (-) | 23 / NA (-) | No / NA (-) | No / NA (-) | No (34) |
| 542-20 | c.542T>G | c.1177A>G | 12 | 17 | Cognitive decline, psychiatric manifestations | LJ | No | 0 / 0 (6) | NA / NA (-) | Spared / Spared (6) | 18 / 18 (6) | No / NA (-) | No / NA (-) | No (18) |
| *542-21 ¥* | *c.542T>G* | ***c.641C>T*** | *-* | *27* | *Presymptomatic* | *A* | *Yes (29)* | *0 / 0 (2)* | *No / No (2)* | *Spared / Spared  (2)* | *5 / 7 (2)* | *No / NA (-)* | *No / NA (-)* | No (29) |
| 542-22 | c.542T>G | **c.465+1G>A** | 11 | 17 | Cognitive decline, psychiatric manifestations | LJ | Yes (18) | 0 / 0 (6) | NA / NA (-) | Yes / Yes (7) | 22 / 22 (7) | Yes / NA (-) | No / NA (-) | No (17) |
| 542-23 | c.542T>G | **c.465+1G>A** | 18 | 26 | Cognitive decline, psychiatric manifestations | A | No | 0 / 0 (8) | NA / NA (-) | Spared / Spared (11) | 22 / 22 (11) | NA / NA (-) | NA / NA (-) | No (26) |
| 542-24 € | c.542T>G | **c.465+1G>A** | 10 | 15 | Cognitive decline, psychiatric manifestations | LJ | Yes (15) | 0 / 1 (6) | NA / No (5) | Yes / Yes (5) | 23 / 23 (5) | NA / No (5) | NA / No (5) | No (15) |
| *542-25 €* | *c.542T>G* | **c.465+1G>A** | *-* | *15* | *Presymptomatic* | *LJ* | *No* | *0 / NA (-)* | *No / NA (-)* | *Spared / NA  (-)* | *9 / NA (-)* | *No / NA (-)* | *Yes / NA (-)* | No (15) |
| 542-26 | c.542T>G | **c.465+1G>A** | 33 | 34 | Cognitive decline, psychiatric manifestations | A | No | 0 / 1 (6) | NA / NA (-) | Spared / NA (-) | 22 / NA (-) | No / NA (-) | No / NA (-) | No (40) |
| 542-27 | c.542T>G | **c.465+1G>A** | 30 | 36 | Cognitive decline | A | No | 0 / 0 (6) | No / NA (-) | Spared / NA (-) | 15 / NA (-) | Yes / NA (-) | No / NA (-) | No (36) |

Clinical characteristics and outcomes of individual patients are shown. Siblings of the same family in this cohort are indicated with an identical symbol next to their patient ID. For GMFC-MLD, cognitive function, involvement of the motor tracts, MRI severity score, demyelinating peripheral neuropathy, and axonal degeneration, the outcome at diagnosis and at latest follow-up is presented. For outcomes at latest follow-up, the disease duration is indicated between brackets and calculated from symptom onset for symptomatic patients at diagnosis or calculated from diagnosis for pre-symptomatic patients at diagnosis and 2 symptomatic patients (257-1, 542-7) with unknown age at symptom onset. Survival is calculated from birth to death for deceased patients and to last clinical evaluation for living patients.

^a^ Functional severity of the second *ARSA* variants is indicated based on Trinidad et al. (2023), with known severe variants in bold, known moderate and mild variants in regular text, and variants with unknown functional severity underscored. Asterisks (*) denote conflicting interpretations of functional severity.

^b^Cognitive function categories are presented as “no” indicating “no to borderline impaired cognitive function”; “mild” indicating “mildly impaired cognitive function”; and “severe” indicating ”severely impaired cognitive function“

^c^Age, disease duration, and survival are given in years

Abbreviations: A = adult; EJ = early-juvenile; HSCT = allogeneic hematopoietic stem cell transplantation; LI = late-infantile; LJ = late-juvenile; NA = not assessed

**Supplementary Table 3: Disease duration at follow-up measurement for untreated patients** **by variable and outcome**

| **Variable (total patient number)  group within variable (patient number)** | | | **Median disease duration in years at the time of measurement** | **iqr** | **range** |
| --- | --- | --- | --- | --- | --- |
| Gross motor function (*n* = 34) | | | 10 | 6 – 16 | 2 – 31 |
|  | GMFC-MLD score 0 (*n* = 20) | | 9 | 6 – 15 | 2 – 31 |
|  | GMFC-MLD score 1 (*n* = 6) | | 10 | 7 – 14 | 3 – 21 |
|  | GMFC-MLD score 2 (*n* = 1) | | 3 | NA | NA |
|  | GMFC-MLD score 3 (*n* = 1) | | 7 | NA | NA |
|  | GMFC-MLD score 4 (*n* = 0) | | NA | NA | NA |
|  | GMFC-MLD score 5 (*n* = 2) | | 19 | NA | NA |
|  | GMFC-MLD score 6 (*n* = 4) | | 15 | 12 – 15 | 4 – 15 |
| Cognitive function (*n* = 11) | | | 9 | 7 – 10 | 4 – 29 |
|  | | No to borderline impaired cognitive function (*n* = 1) | 4 | NA | NA |
|  | | Mildly impaired cognitive function  (*n* = 2) | 9 and 14 | NA | NA |
|  | | Severely impaired cognitive function (*n* = 8) | 9 | 8 – 14 | 4 – 29 |
| Involvement of the motor tracts (*n* = 21) | | | 10 | 7 – 11 | 1 – 31 |
|  | | Relatively spared (*n* = 15) | 10 | 7 – 13 | 1 – 31 |
|  | | Not spared (*n* = 6) | 9 | 5 – 11 | 3 – 15 |
| MRI severity (*n* = 21) | | | 10 | 7 – 11 | 1 – 31 |
|  | | c.256C>T (*n* = 7) | 10 | 5 – 13 | 1 – 27 |
|  | | c.257G>A (*n* = 4) | 10 | 9 – 11 | 7 – 15 |
|  | | c.542T>G (*n* = 10) | 10 | 7 – 11 | 3 – 31 |
| Presence of peripheral neuropathy (*n* = 17) | | | 11 | 4 – 15 | 1 – 31 |
|  | | Demyelinating neuropathy (*n* = 6) | 12 | 11 – 17 | 3 – 31 |
|  | | No demyelinating neuropathy (*n* = 11) | 9 | 4 – 14 | 1 – 27 |
|  | | Axonal degeneration (*n* = 6) | 12 | 6 – 14 | 2 – 18 |
|  | | No axonal degeneration (*n* = 11) | 11 | 6 – 17 | 1 – 31 |

This table shows the median disease duration with iqr and range in years at the time of measurement of a variable at follow-up for untreated patients in total and per compared groups. Disease duration was calculated from symptom onset for symptomatic patients and from diagnosis for 1 pre-symptomatic patient who was diagnosed at the age of 42 years and was still pre-symptomatic after 4 years.

Abbreviations: GMFC-MLD = Gross Motor Function Classification in metachromatic leukodystrophy, iqr = interquartile range; NA = not assessed.
